# Phylogeography, Transmission, and Viral Proteins of Nipah Virus

**DOI:** 10.1007/s12250-018-0050-1

**Published:** 2018-10-11

**Authors:** Bangyao Sun, Lijia Jia, Bilin Liang, Quanjiao Chen, Di Liu

**Affiliations:** 1 Wuhan Institute of Virology, Chinese Academy of Sciences, Wuhan, 430071 China; 2 University of Chinese Academy of Sciences, Beijing, 100049 China

**Keywords:** Nipah virus, Viral transmission, Geographical distribution, Protein structure

## Abstract

Nipah virus (NiV), a zoonotic paramyxovirus belonging to the genus *Henipavirus*, is classified as a Biosafety Level-4 pathogen based on its high pathogenicity in humans and the lack of available vaccines or therapeutics. Since its initial emergence in 1998 in Malaysia, this virus has become a great threat to domestic animals and humans. Sporadic outbreaks and person-to-person transmission over the past two decades have resulted in hundreds of human fatalities. Epidemiological surveys have shown that NiV is distributed in Asia, Africa, and the South Pacific Ocean, and is transmitted by its natural reservoir, *Pteropid* bats. Numerous efforts have been made to analyze viral protein function and structure to develop feasible strategies for drug design. Increasing surveillance and preventative measures for the viral infectious disease are urgently needed.

## Introduction

In February 2018, Nipah Virus (NiV) infection was listed as a priority disease posing a public health risk by the World Health Organization (http://www.who.int/blueprint/en/). NiV was named after Kampung Sungai Nipah (Nipah River Village) in Malaysia, where it was first isolated in 1998, before its subsequent spread into Singapore via exported pigs in 1999, leading to the abattoir worker infections (CDC 1999a, b; Paton *et al.* 1999; Epstein *et al.* 2006). In 2001, human cases of NiV infection were discovered independently in India and Bangladesh, and since then, infections have been observed annually in Bangladesh, and human-to-human transmission through direct contact with infected individuals is common (Hsu *et al.* 2004; Chadha *et al.* 2006a, b). In 2007, an NiV outbreak occurred in India, killing five people (Arankalle *et al.* 2011). In 2018, NiV infection was ongoing in Kerala, India, with 16 cases succumbed (Paul 2018). In 2014, a serious illness most probably caused by NiV was reported in several people after contact with infected horses or patients in the Philippines (Ching *et al.* 2015). NiV infection can cause fever and encephalitis in humans and a neurological and respiratory syndrome in pigs or horses (Lee *et al.* 1999; CDC 1999a, b). To date, over 600 human cases of NiV infection have been reported in South Asia and South-East Asia, with fatality ranging from 40% to 70%, accordingly it poses a major threat to human health (Clayton 2017).

**Fig. 1:**
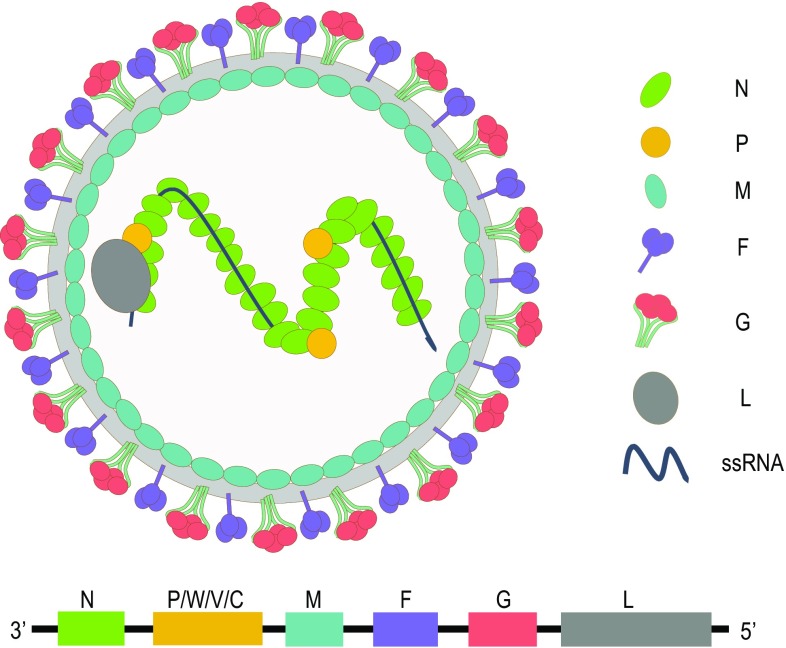
Schematic representation of the viral structure (upper panel) and genome organization (lower panel). Different genes or proteins are indicated in different colors.

Belonging to the genus *Henipavirus* [the other pathogenic member of the genus is Hendra virus (HeV), reviewed in (Middleton 2014; Enchéry and Horvat 2017)] of the family *Paramyxoviridae* (Chua *et al.* 2000), NiV is classified as a Biosafety Level-4 (BSL-4) pathogen due to its high pathogenicity and the lack of any effective treatments or vaccines (Wit and Munster 2015; Angeletti *et al.* 2016). The NiV genome consists of a negative-sense, single-stranded RNA of approximately 18.2 kb, encoding six structural proteins, nucleoprotein (N), phosphoprotein (P), matrix protein (M), fusion protein (F), attachment glycoprotein (G), and the large protein or RNA polymerase protein (L). In addition, the *P* gene encodes three nonstructural proteins by RNA editing (V and W proteins) or an alternative open reading frame (C protein) (Wang *et al.* 2001) (Fig. 1).

NiV has a wide range of hosts, from its natural reservoir *Pteropid* bats to humans, horses, dogs, cats, cows, and pigs (Calisher *et al.* 2006; Halpin *et al.* 2011; Weatherman *et al.* 2018). Close contact with infected patients (Tan and Tan 2001) or domestic animals (e.g., pigs and horses) plays an important role in the spread of NiV (Clayton 2017). Furthermore, palm sap is also currently regarded as a crucial NiV transmission medium in Bangladesh (Luby *et al.* 2006; Nahar *et al.* 2010; Rahman *et al.* 2012). Intraspecific transmission (in bats, pigs, and horses) is also possible via saliva, urine or secretions upon high density populations of animals (Middleton *et al.* 2007; Weatherman *et al.* 2018). In this review, an overview of recent studies on the geographical and phylogenetic properties, transmission, and protein structure and function of NiV is provided.

## Geographical Distribution of Nipah Virus

**Fig. 2:**
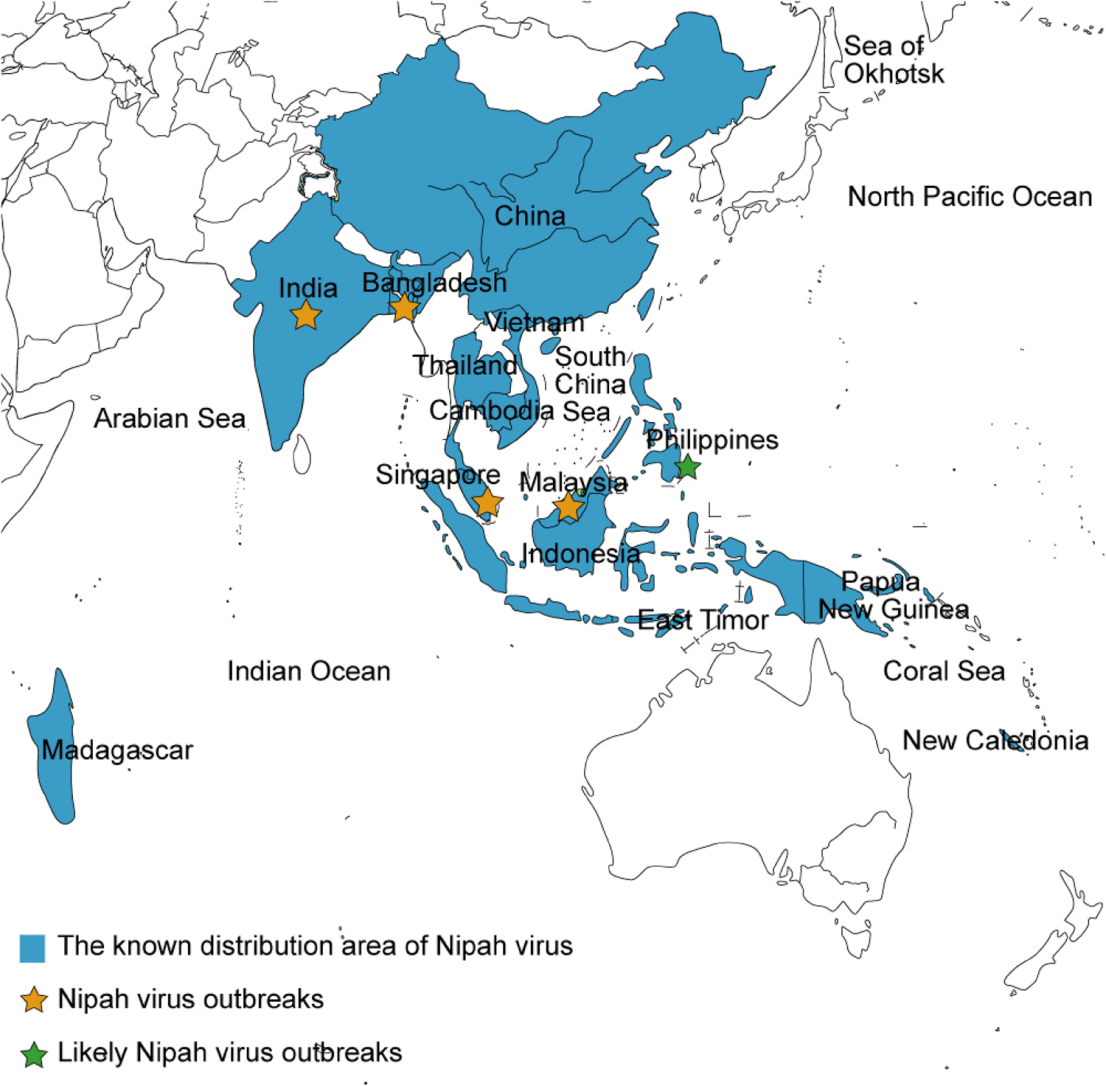
Summary of the known geographical distribution of Nipah virus in the world. Yellow stars represent reported Nipah virus outbreaks and green star shows the likely presence of Nipah-like virus.

Since it emerged in Malaysia in 1998, NiV caused a series of outbreaks in Singapore, India, and Bangladesh (Clayton 2017; Enchéry and Horvat 2017) (Fig. 2), which has caused hundreds of human deaths and thereby represents a great challenge to public health. Numerous efforts have been made to trace the origin, distribution, and probable transmission route of NiV in nature. For example, in Bangladesh, where humans are frequently infected by NiV, retrospective investigations combined with the collect of biological samples from patients or contaminated environment have been conducted to evaluate potential risk factors and to develop a feasible strategy for prevention and control (Hsu *et al.* 2004; Gurley *et al.* 2007; Rahman *et al.* 2012). Additionally, NiV surveillance in areas where no NiV outbreaks have been reported is ongoing. Extensive studies and sample collections (swabs, sera, saliva, and urine) and analyses from bats have indicated that in addition to the countries where NiV outbreaks have occurred, NiV is also distributed in China (Yan *et al.* 2008), Vietnam (Hasebe *et al.* 2012), Thailand (Supaporn *et al.* 2005), Cambodia (Reynes *et al.* 2005), Indonesia (Sendow *et al.* 2010), East Timor (Breed *et al.* 2013), Madagascar (Iehlé *et al.* 2007), New Caledonia (Enchéry and Horvat 2017) and Papua New Guinea (Breed *et al.* 2010; Field *et al.* 2013) (Fig. 2). In addition, anti-NiV neutralization antibody test of the serum from people involved in hunting bats as bushmeat revealed evidence of NiV spillover (Pernet *et al.* 2013), which emphasizes the significance of further NiV monitoring.

## Hosts and Transmission of Nipah Virus

**Fig. 3:**
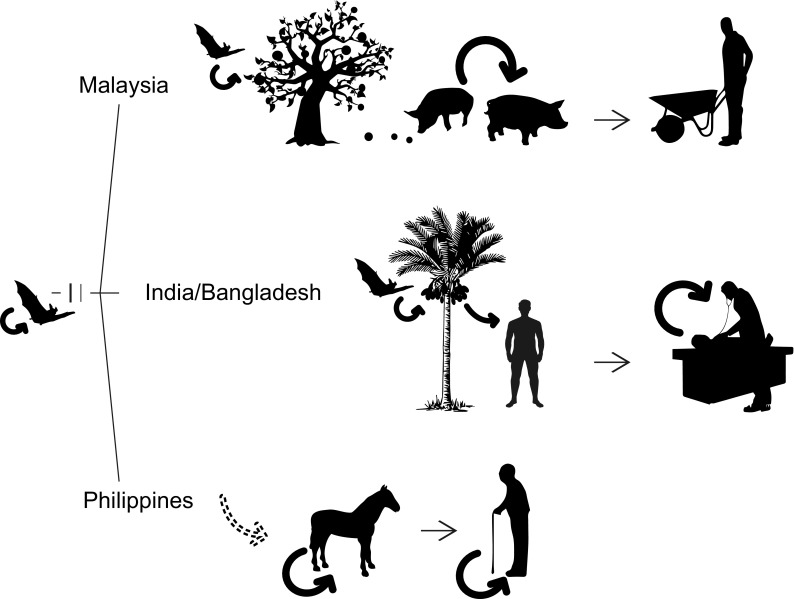
Schematic representation of transmission routes for Nipah virus. In Malaysia, the fruit trees where fruit bats reside are in proximity to pig farms and domestic pigs infected by NiV via contact with materials contaminated by bats, and NiV is subsequently transmitted to humans by direct contact. In India or Bangladesh, persons infected by NiV after consuming the date palm sap contaminated by bat saliva or urine, followed by person-to-person transmission by close contact. In the Philippines, people were infected by consuming horsemeat or contact with infected horses, and then healthy individuals were infected after contact with patients.

Understanding susceptible hosts and routes for the spread of viral disease raises knowledge to curb epidemics. Bats are the second largest order of mammals after rodents (Moratelli and Calisher 2015; Ming and Dong 2016), and harbor in excess of 200 types of viruses, including many highly pathogenic to humans (e.g., rabies, Ebola, severe acute respiratory syndrome (SARS), NiV, HeV) (Li *et al.* 2005; Calisher *et al.* 2006). NiV circulates within bat populations via close mutual contact when bats crowd together (Middleton *et al.* 2007). NiV transmission from bats to humans is through two main pathways, i.e., intermediate hosts (pigs and horses) and food-borne transmission via date palm sap contaminated with the saliva or urine of fruit bats (Enchéry and Horvat 2017) [reviewed in (Clayton 2017)]. A retrospective study in Malaysia found that workers show severe influenza-like symptoms after slaughtering NiV-infected swine (Hsu *et al.* 2004). In the Philippines, people were infected by butchering horses or consuming horsemeat (Ching *et al.* 2015). No cases of person-to-person spread have been found in Malaysia or Singapore, but in the Philippines, direct human-to-human virus transmission has been reported (Ching *et al.* 2015). In India, human-to-human transmission of NiV was discovered in 2001. In a case of NiV infection in a human in 2007, date palm sap contaminated by bats was considered to mediate NiV spillover from bats to humans (Chadha *et al.* 2006a, 2006b; Arankalle *et al.* 2011). In Bangladesh, where people consume palm sap, frequent infection by NiV and person-to-person NiV transmission has occurred. Recently, it was demonstrated that Syrian hamster infection could occur after drinking artificial palm sap mixed with NiV (Wit *et al.* 2014) and infrared camera monitoring showed that bats frequently fly around or directly contact palm sap trees to urinate or defecate (Khan *et al.* 2010; Rahman *et al.* 2012). These findings provide further support for palm sap-mediated bat-to-person transmission, despite the lack of Nipah virus detection in natural date palm sap until now (Fig. 3). Measures have been taken to prevent bat access to sap using bamboo skirts or lime smudged on date palm trees. Further steps to prevent the transmission of NiV infection are necessary.

## Viral Genomics and Phylogenetics

**Fig. 4:**
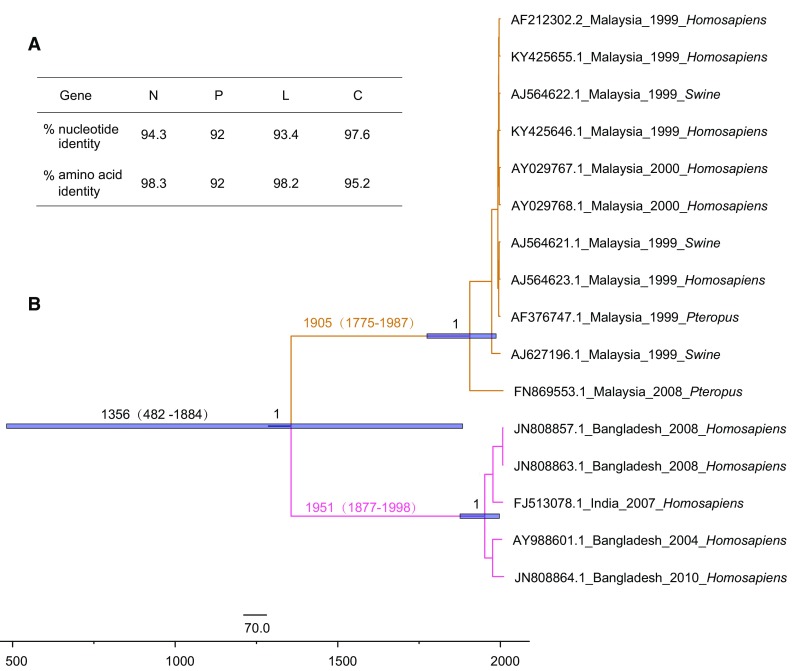
Genomic and phylogenetic analyses of Nipah virus. **A** Comparisons of both nucleotide sequence and amino acid sequence identity between NiV-Bangladesh and NiV-Malaysia. **B** Maximum clade credibility (MCC) tree of Nipah virus genomes. The tree was performed by using BEAST package 2.4.8 with the HKY SRD06 model, under an uncorrelated relaxed clock, and the exponential growth demographic model. NiV-Bangladesh is represented in pink and NiV-Malaysia is in brown. The purple bars represent the 95% highest posterior density intervals of the estimation of the dates.

Genomic and amino acid differences may explain differences in viral pathogenicity and virulence between isolates in Bangladesh and Malaysia, particularly given the contributions of the N, P and L proteins to viral replication and transcription and the role of the nonstructural protein C in the virulence of NiV (see “Viral Protein Function and Structure” section). In sequence analyses (Harcourt *et al.* 2005), nucleotide sequence identity between the genomes of NiV-Bangladesh and NiV-Malaysia was only 91.8%, with an uneven distribution of differences throughout the genome. The amino acid sequence identity between NiV-Bangladesh and NiV-Malaysia proteins were all greater than 92%. Furthermore, the nucleotide sequence identity of *N*, *P*, *L*, and *C* genes were 94.3%, 92.0%, 93.4%, and 97.6%, respectively, with corresponding amino acid sequence identity of 98.3%, 92%, 98.2%, and 95.2%, respectively (Harcourt *et al.* 2005) (Fig. 4A). These findings provided a basis for further investigations of the biological characteristics of NiV.

Based on the time-scaled tree constructed using the currently available viral genomes, the time to the most recent common ancestor (tMRCA) of NiV could be dated to 1356 (95% highest posterior density, 95% HPD: 482—1884). The strains were divided into two lineages (NiV-Bangladesh, n = 4, and NiV-Malaysia, n = 11), with different clinical features and transmission routes in Bangladesh and Malaysia (Fig. 4B). Due to the limited viral genomes available, the detailed divergent time of Bangladesh and Malaysia lineages needs further investigation. More frequent person-to-person contact and more severe respiratory disease have been observed in Bangladesh than in Malaysia (Goh *et al.* 2000; Chong *et al.* 2008; Hossain *et al.* 2008), consistent with the higher level of viral replication in ferrets for NiV-Bangladesh than NiV-Malaysia (Clayton *et al.* 2012). However, a recent study suggested that social and environmental factors impact the spread of NiV (Clayton and Marsh 2014; Clayton *et al.* 2016). In Malaysia, no person-to-person transmission was reported when NiV emerged in 1998 and NiV strains derived from humans were isolated in 1999–2000 (Fig. 4B), whereas, in Bangladesh, NiV has appeared nearly annually since 2001 along with significant human-to-human transmission. Thus, comparative genomics and reverse genetics approaches are required to uncover the different features between NiV-Bangladesh and NiV-Malaysia.

## Viral Protein Function and Structure

Nipah virus has an approximately 18.2 kb genome encoding six structural proteins and three nonstructural proteins. The viral ribonucleocapsid (RNP) surrounded by the viral envelope consists of its genome and the N protein, which is essential for the viral life cycle as a template for RNA-dependent RNA-polymerase (RdRp), composed of polymerase L and a polymerase cofactor P (Diederich and Maisner 2007; Cox and Plemper 2017). Within the RNP, N is responsible for viral genome wrapping and facilitates viral replication and transcription (Lee *et al.* 2012). The synthesis of viral mRNA is catalyzed by L and P (Morin *et al.* 2013), and the latter also inhibits interferon signaling via host STAT-1 (Lo *et al.* 2012) and acts as a chaperone of N^0^ (the unassembled form of N) to prevent it nonspecific binding to host RNA (Habchi and Longhi 2012). The M protein contributes to viral assembly and release (Dietzel *et al.* 2015). G and F are two important surface glycoproteins of NiV; the former induces viral attachment to two cellular receptors, ephrin-B2 and ephrin-B3, despite a lack of hemagglutination or neuraminidase activity (Bonaparte *et al.* 2005; Negrete *et al.* 2005, 2006; Bishop *et al.* 2007). And this subsequently triggers F-mediated membrane fusion between virus and host cells (Bossart *et al.* 2002; Tamin *et al.* 2002). The nonstructural protein C participates in the host immune response and serves as a virulence factor (Mathieu *et al.* 2012).

**Fig. 5:**
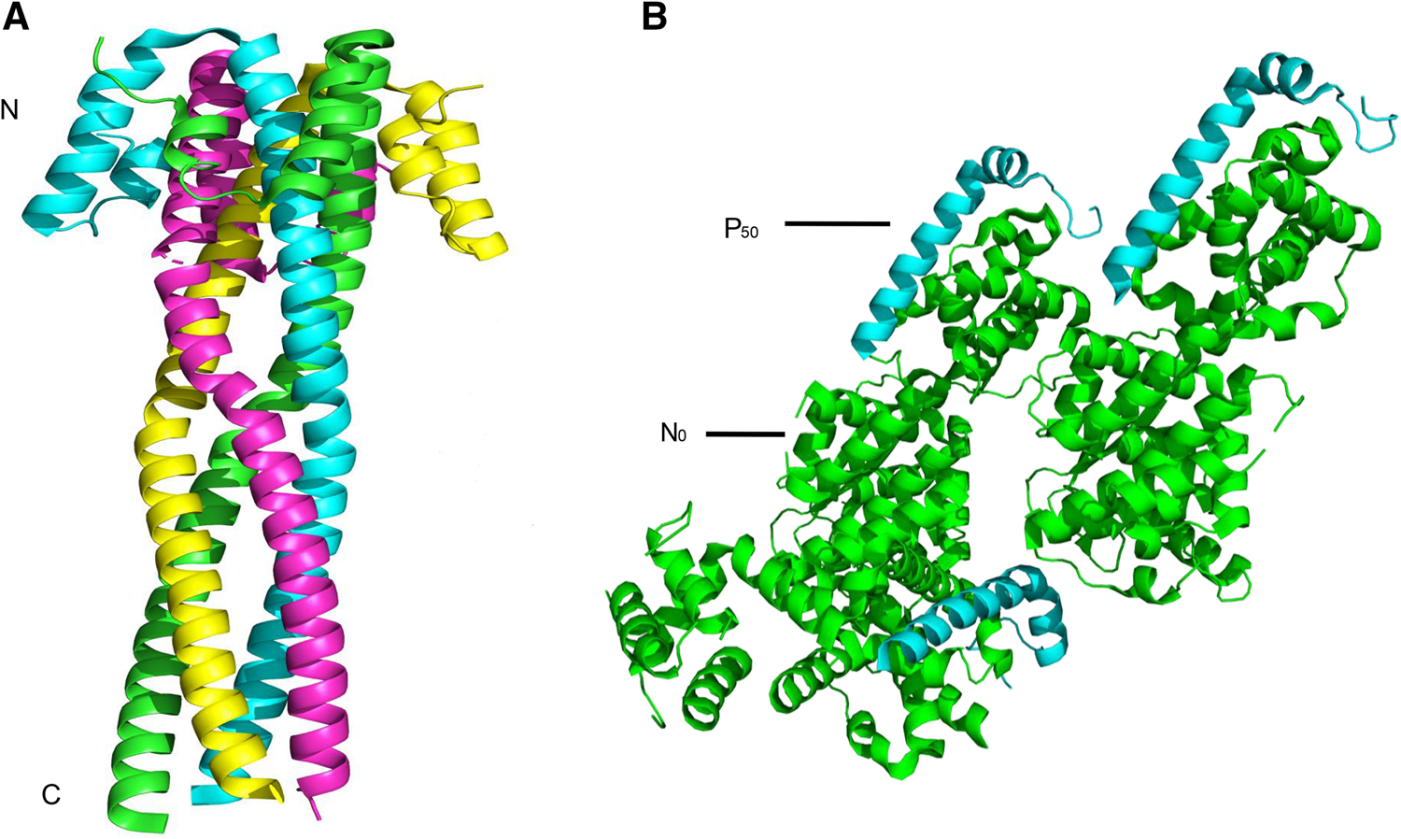
Crystal structures of P and the N^0^-P complex. **A** Cartoon representation of NiV-P (Protein Data Bank accession number 4N5B). Four chains are indicated in four different colors. N and C termini are shown. **B** Structure of three heterodimers of the N^0^-P complex (PDB: 4CO6). P_50_ is shown in cyan and N^0^ is in green.

Elucidating the viral structure is regarded as a promising approach for antiviral drug design, and several crystal structures of NiV proteins have been reported. The crystal structure of the P protein is a tetramer with a parallel coiled coil (Fig. 5A), while the N^0^-P complex, whose binding site is located in residues 1–50 (P_50_) of the N-terminal domain of P, is characterized by an asymmetric pea-like form composed of three heterodimers. N^0^ remains an open conformation in the complex due to P-mediated inhibition of the polymerization of N (Bruhn *et al.* 2014; Yabukarski *et al.* 2014) (Fig. 5B).

**Fig. 6:**
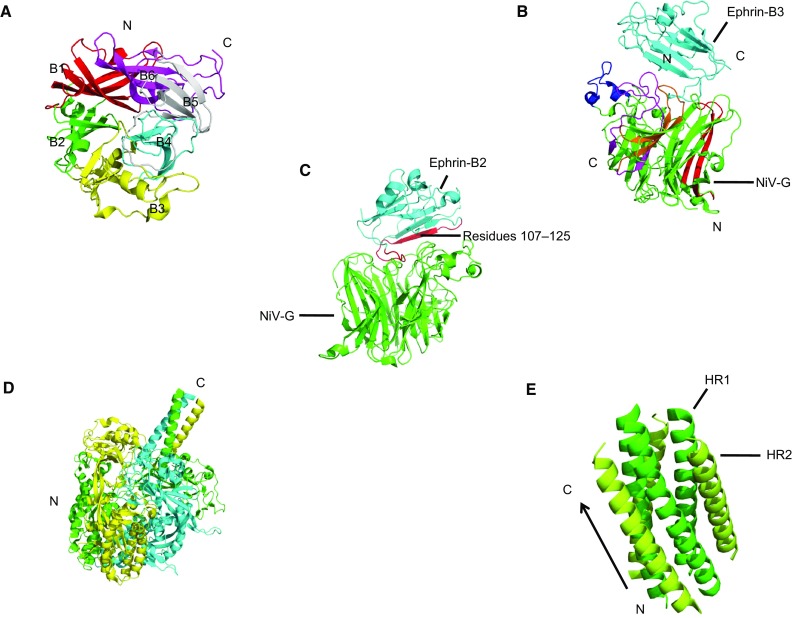
Crystal structures of G and F protein. **A** Cartoon representation of NiV-G (PDB: 3D11) in the receptor-unbound state. Six blades are represented as follows: B1 in red, B2 in green, B3 in yellow, B4 in cyan, B5 in gray and B6 in magenta. N and C termini are shown in the figure. The disulfide bond (C181–C601) is not shown. **B** Structure of the G/Ephrin-B3 complex (PDB: 3D12). The upper Ephrin-B3 is labeled in cyan and the lower G is in green. Four G loops are indicated as following, B1S2–B1S3 in red, B3H2–B3H3 in blue, B4S4–B5S1 in magenta, and B5S2–B5S3 in orange. **C** An overview of the G/Ephrin-B2 complex structure (PDB: 2VSM). NiV-G and Ephrin-B2 are shown in green and cyan, respectively, and residues 107–125 is in green. **D** The structure of NiV-F pre-fusion (PDB: 5EVM). Three chains of F trimers are indicated in green, yellow, and cyan, and the hexamer form of F trimers is not represented. **E** Cartoon representation of the fusion core (PDB: 1WP7). HR1 is indicated in green and HR2 is in lemon with N and C termini.

The extracellular region of the attachment glycoprotein G, which is a tetrameric type II membrane protein, is a disk-like β-propeller with six blades (B1–B6) encircling the center in its receptor-unbound status and changes its form in the G/Ephrin-B3 complex. Each blade module in the β-propeller enhanced by a disulfide bond (C181–C601) contains a four-stranded (strands S1–S4) antiparallel β-sheet (Fig. 6A). The structure of the G/Ephrin-B3 complex is elongated with a heterodimeric assembly compared to G (Xu *et al.* 2008). Ephrin-B3 attaches to the upper face of the G β-propeller and their interaction is mediated by several G loops, including B1S2–B1S3, B3H2–B3H3, B4S4–B5S1, and B5S2–B5S3 (Fig. 6B). The crystal structure of the G/Ephrin-B2 complex is characterized by a capacious protein–protein interface containing crucial residues 107–125 on Ephrin-B2 (Bowden *et al.* 2008) (Fig. 6C). F, a typical trimeric class I membrane protein, is synthesized as an immature precursor (called F_0_) and is then cleaved by cellular protease into F_1_ and F_2_ subunits linked by a disulfide bond. Two heptad-repeat regions (HR1 and HR2) in F_1_ contribute to the membrane merger (Michalski *et al.* 2000; Tamin *et al.* 2002). In the pre-fusion form of the ectodomain of F, six copies of F trimers assemble a hexamer around a central axis (Xu *et al.* 2015), which confers the stability of pre-fusion F (Fig. 6D). During subsequent membrane fusion, the conformation of two HR domains changes and a six-helix bundle, called a fusion core, takes shape to facilitate viral penetration after the merger, characterized by three parallel HR1 domains surrounded by three anti-parallel HR2 domains (Xu *et al.* 2004; Lou *et al.* 2006) (Fig. 6E). Given that only supportive treatments are available in hospital setting for NiV [detailed information about viral tropism, vaccines and antiviral strategies of NiV was reviewed in references (Broder *et al.* 2016; Ang *et al.* 2018)], these results provide potential targets for drug or vaccine design.

## Conclusion

Knowing the geographic distribution and transmission of a virus is the priority for the control of infection and resolving the structure and function of viral protein is the basis for anti-viral drug development. In this review, we are focusing on these aspects of the NiV. As a huge natural reservoir of viruses, including NiV, bats have been under renewed interest. Bats appear asymptomatic when infected by many viruses and play a pivotal role in viral spillover. Continually emerging and reemerging viruses from bats have been reported. In 2012, a novel rubula-like paramyxovirus from fruit bats was found to be responsible for a series of severe clinical symptoms appearing on a female wildlife biologist who performed a 6-week field exploration in South Sudan and Uganda (Albariño *et al.* 2014). In 2017, a huge gene pool of SARS-like coronaviruses was found in horseshoe bats in a cave in Yunnan province, China, which indicated the close relationship between those isolates and SARS coronavirus (Hu *et al.* 2017). Recently, a novel bat-originated coronavirus, swine acute diarrhea syndrome coronavirus (SADS-CoV), which led to more than 24,000 piglet deaths at four pig farms in Guangdong province, China was reported (Zhou *et al.* 2018). In particular, a similar transmission pathway between SADS-CoV and NiV (bats to pigs) occurs, although no human infection was found. More exposure to areas of bat movement augments extremely risk of infecting bat-derived viral diseases. Unfortunately, human activities are altering the frequency of contact with bats. For instance, deforestation in tropical zones forces bats to migrate from their habitats to human areas (Daszak *et al.* 2001) and many bats are hunted for consumption or so-called harmfulness (Enchéry and Horvat 2017). Therefore, continuous surveillance, reducing human activities that promote contact with bats, and enhancing scientific research will help the prevention and control of bat-derived viral infectious disease. Given limited viral genomes available, restrictions on investigating the origin and evolution of Nipah virus have been imposed; therefore, continuous epidemiological surveillance must be strengthened in the future. In addition, several structures of viral proteins remain unknown; accordingly, it is necessary to increase basic research efforts on protein structures and functional analyses of viral proteins to provide data for antiviral drug and vaccine development.
